# Significant treatment effect of adjunct music therapy to standard treatment on the positive, negative, and mood symptoms of schizophrenic patients: a meta-analysis

**DOI:** 10.1186/s12888-016-0718-8

**Published:** 2016-01-26

**Authors:** Ping-Tao Tseng, Yen-Wen Chen, Pao-Yen Lin, Kun-Yu Tu, Hung-Yu Wang, Yu-Shian Cheng, Yi-Chung Chang, Chih-Hua Chang, Weilun Chung, Ching-Kuan Wu

**Affiliations:** Department of Psychiatry, Tsyr-Huey Mental Hospital, Kaohsiung Jen-Ai’s Home, Taiwan, No.509, Fengping 1st Rd., Daliao Dist, Kaohsiung City, 831 Taiwan; Department of Neurology, E-Da Hospital, Kaohsiung, Taiwan; Department of Psychiatry, Kaohsiung Chang Gung Memorial Hospital and Chang Gung University College of Medicine, Kaohsiung, Taiwan; Center for Translational Research in Biomedical Sciences, Kaohsiung Chang Gung Memorial Hospital, Kaohsiung, Taiwan

**Keywords:** Psychiatry, Adjunct therapy, Schizophrenia, Music therapy, Alternative therapy

## Abstract

**Background:**

Music therapy (MT) has been used as adjunct therapy for schizophrenia for decades. However, its role is still inconclusive. A recent meta-analysis demonstrated that MT for schizophrenic patients only significantly benefits negative symptoms and mood symptoms rather than positive symptoms. In addition, the association between specific characteristics of MT and the treatment effect remains unclear. The aim of this study was to update the published data and to explore the role of music therapy in adjunct treatment in schizophrenia with a thorough meta-analysis.

**Methods:**

We compared the treatment effect in schizophrenic patients with standard treatment who did and did not receive adjunct MT through a meta-analysis, and investigated the clinical characteristics of MT through meta-regression.

**Results:**

The main finding was that the treatment effect was significantly better in the patients who received adjunct MT than in those who did not, in negative symptoms, mood symptoms, and also positive symptoms (all *p* < 0.05). This significance did not change after dividing the patients into subgroups of different total duration of MT, amounts of sessions, or frequency of MT. Besides, the treatment effect on the general symptoms was significantly positively associated with the whole duration of illness, indicating that MT would be beneficial for schizophrenic patients with a chronic course.

**Conclusions:**

Our meta-analysis highlights a significantly better treatment effect in schizophrenic patients who received MT than in those who did not, especially in those with a chronic course, regardless of the duration, frequency, or amounts of sessions of MT. These findings provide evidence that clinicians should apply MT for schizophrenic patients to alleviate disease severity.

**Electronic supplementary material:**

The online version of this article (doi:10.1186/s12888-016-0718-8) contains supplementary material, which is available to authorized users.

## Background

Schizophrenia is one of the most serious psychiatric diseases worldwide. It causes significant dysfunction in multi-dimensions of personal function of the patients, with a wide range of clinical symptoms. In general, the symptoms of schizophrenia can be classified as positive symptoms, negative symptoms, and general psychopathology [1]. In addition, patients with schizophrenia also often have comorbid mood symptoms, either in the form of a manic mood or depression. Furthermore, risk factors associated with schizophrenia such as violence and suicide have been found to be correlated with the specific symptoms of schizophrenia. Among them, the risk of violence has been reported to be correlated with positive symptoms [2], and the risk of suicide has been reported to be correlated with negative symptoms and mood symptoms [3, 4].

Music therapy has been used in the treatment of schizophrenia for decades, and it is often used as adjunct therapy to medication. It can take the format of individual therapy [5], large group therapy [6], or a combination of individual and group therapy [7] through either “passive listening” [8–10] or “active participation” [6, 7]. Every form of music therapy has an effect in schizophrenic patients to a certain extent. However, most studies investigating the treatment effect of music therapy have focused on the effect on negative and mood symptoms only [6, 7, 9–13].

Recently, there is one huge review article discussing the clinical application of music therapy in different disease entity, including neoplasm, disease of central nervous system, psychiatric illness, and so on [14]. In this article, the authors reviewed the evidences of music therapy in the schizophrenic patients mainly according to one recent meta-analysis conducted by the Mössler and the colleagues (2011) [15]. In this meta-analysis conducted by Mössler and the colleagues, the authors suggested that the application of music therapy in schizophrenic patients would be beneficial with regards to negative symptoms and mood symptoms. On the other hand, because of a lack of evidence, the role of music therapy in the treatment of positive symptoms in schizophrenic patients is inconclusive. However, positive symptoms have been proven to be highly associated with the risk of violence [2], which contributes to the stigmatization of patients with schizophrenia [16]. Furthermore, the report by Mössler et al. (2011) did not provide further information about the association between the treatment effect of music therapy and the specific characteristics of music therapy applied in schizophrenic patients.

In order to explore (1) the treatment effect of music therapy in patients with schizophrenia, and (2) the role of specific characteristics of music therapy in the treatment effect, we conducted this meta-analysis to evaluate the treatment effect of music therapy on the specific subscale symptoms of schizophrenia, including the overall disease severity of schizophrenia, positive symptoms, negative symptoms, general psychopathology, and mood symptoms. We performed meta-regression analysis to evaluate the association between the treatment effect of music therapy and the clinical variables or specific characteristics of music therapy, including frequency, duration of each session, total number of sessions of music therapy, and total duration of music therapy.

## Methods

### Database schema and implementation

**Fig. 1:**
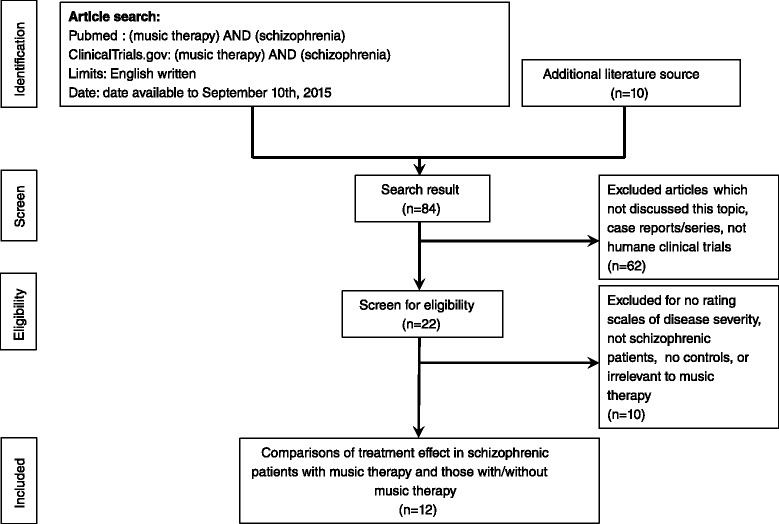
Flowchart of the selection strategy and inclusion/exclusion criteria for the current meta-analysis

A systematic literature search was conducted by two independent psychiatrists (Wang, H.Y. and Tu, K.Y.) through PubMed. If there was an inconsistent selection and lack of agreement, a final decision was made by consensus. The search was performed using the key words “(music therapy) AND (schizophrenia)” for all articles written in English available unto September 10th, 2015. Initially, all of the search results meeting the inclusion criteria were collected, and the titles and abstracts were screened by Wang, H.Y. and Tu, K.Y. When there was disagreement on eligibility, we reached agreement through consensus. In current study protocol, the definition of music therapy had two forms of definition; the wide definition was that all the music applied in the treatment of patients, and the narrow one was that the music therapy should be arranged under specific settings, for example, group therapy, individual therapy, active participation, or directed by a specialists. All studies that were not related to music therapy in schizophrenic patients were excluded. Using the following inclusion criteria, we thoroughly screened the studies: (1) articles discussing comparisons of the treatment effect in patients with schizophrenia treated with music therapy and those with/without placebo; (2) articles on clinical trials in humans; and (3) case-controlled trials, either randomized or non-randomized. The exclusion criteria were (1) case reports or series; and (2) non-clinical trials. The screening and search protocol is shown in Fig. 1. We used Jadad scores to investigate the quality of the clinical trials included in this meta-analysis [17].

### Data extraction

The primary outcome was disease severity rating scale, which varied in every study and included the Brief Psychiatric Rating Scales (BPRS) [18] , the Positive And Negative Symptoms Scales (PANSS) [1], the Scale for the Assessment of Negative Symptoms (SANS) [19], and the Self-rating Depression Scales (SDS) [20]. All of the primary outcomes and other clinical variables were extracted from all of the studies included in the current meta-analysis. When data were not available, we tried to contact the authors to acquire the original data. Among the rating scales, we gave priority to the PANSS rather than the BPRS, SANS, and SDS, because it is more specific to the setting of schizophrenia.

### Meta-analytic methods and quality of control

All of the effect sizes (ESs), which indicated changes in disease severity rating scales in the included studies, were set as the standardized mean difference based on Hedges’ adjusted *g*. We defined an ES greater than 0 as indicating “greater improvements in the primary outcome in schizophrenic patients who received music therapy than in patients treated with/without a placebo”. We tried to derive the ESs from other statistical parameters such as the *t* or *p* value with the sample size when the disease severity rating scales or the original data from the authors were unavailable. All of the ESs were synthesized using a random effects model for every meta-analysis.

All of the meta-analytic procedures were performed using Comprehensive Meta-Analysis software, version 2 (Biostat, Englewood, NJ, USA). We considered the analysis to be statistically significant when a two-tailed *p* value was <0.05. We used Q statistics, their related p-value, and the *I*^*2*^ statistic to investigate the heterogeneity of each study. We investigated publication bias through funnel plots, and Egger’s regression analysis was used to test statistically for the significance of any possible publication bias [21]. We also performed subgroup meta-analysis or meta-regression analysis using the unrestricted maximum likelihood method to examine possible sources of heterogeneity and to investigate the possible confounding effects of clinical variables such as age, gender, duration of illness, number of previous hospitalizations, chlorpromazine equivalence, frequency of music therapy, total duration of music therapy, total number of sessions of music therapy, duration of each session of music therapy, disease severity, and “whole duration of illness”. We defined whole duration of illness as the period from the onset of psychosis to the time when the patients entered the study. Furthermore, we performed subgroup meta-analysis of positive symptoms, negative symptoms, general psychopathology, and mood symptoms for the treatment effect of music therapy. In addition, to investigate whether or not the study design contributed to a different treatment effect, we performed subgroup meta-analysis to evaluate the treatment effect of music therapy in trials with randomized control trials (RCTs) or non-randomized control trials (non-RCTs). Besides, we also perform subgroup meta-analysis to investigate the possible confounding effect of different strategy of music therapy on the treatment effect. These meta-analytic procedures fulfilled the criteria of Preferred Reporting Items for Systematic reviews and Meta-Analyses (PRISMA) (Additional file 1) [22]. About the ethics of current study, it was not necessary for the ethical approval because that we would not deal with the patients’ detailed data. Furthermore, it was impossible for us to contact all the patients for the informed consent.

## Results

### Studies included in each meta-analysis

After screening, a total of 12 articles remained for meta-analysis (Table 1) [5–7, 9–13, 23–26]. One of these studies used the SDS only which is less specific to the disease severity of schizophrenia [10], and thus we included this study in the meta-analysis of the treatment effect of music therapy on the mood symptoms of schizophrenic patients only. The quality of the clinical trials was rated with an average Jadad score of 1.58 (Additional file 2: Table S1).

**Table 1:** Summary of characteristics of studies in current meta-analysis

Study	Diagnostic criteria	Diagnosis	Comparison	N	Dropout rate	Mean age (years)	Gender (%female)	Chlorpromazine equivalence (mg/day)	Severity	Primary outcome	Subscale	Trials	Country
Gold, C. (2013)^11^	ICD-10	Psychotic disorder Affective disorder	Music therapy Control	72 72	27.8 33.3	34.0 ± 11.3	47.9	n/a	n/a	SANS	negative symptoms	Prospective, SB, RCT	Norway
Lu, S.F. (2013)^14^	DSM-IV	Schizophrenia	Music therapy Control	38 42	7.9 4.8	52.02 ± 7.6	26.3	530.2 ± 146.4	mild	PANSS	positive, negative, general psychopathology, mood symptoms	Prospective, SB, RCT	Taiwan
Peng, S.M. (2010)^15^	DSM-IV-TR	Schizophrenia	Music therapy Control	29 30	9.4 14.3	36.0 ± 7.2	45.8	n/a	mild	BPRS	positive, negative, mood symptoms	Prospective, RCT	Taiwan
Li, Y.M. (2007)^10^	CCMD-3	Schizophrenia	Music therapy Control	30 30	0.0 0.0	32.0 ± 12.0	0	n/a	n/a	SDS	mood symptoms	Prospective, RCT	China
Ulrich, G. (2007)^13^	ICD-10	Schizophrenia Schizoaffective	Music therapy Control	21 16	23.8 31.3	37.8 ± 9.4	45.9	456.7 ± 313.9	mild	SANS	negative symptoms	Prospective, DB, RCT	Germany
Talwar, N. (2006)^5^	ICD-10	Schizophrenia	Music therapy Control	33 48	15.2 14.6	37.4 ± 11.4	25.9	453.8 ± 376.0	mild	PANSS	positive, negative, general psychopathology	Prospective, SB, RCT	UK
He, F.R. (2005)^9^	CCMD-3	Schizophrenia	Music therapy Control	30 30	n/a	35.0 ± 8.0	15.0	n/a	n/a	SANS	negative symptoms	Prospective, RCT	China
Wen, S.R. (2005)^12^	CCMD-3	Schizophrenia	Music therapy Control	16 14	0.0 0.0	n/a	30.0	n/a	moderate	BPRS	mood symptoms	Prospective, RCT	China
Hayashi, N. (2002)^16^	DSM-IV	Schizophrenia Schizoaffective	Music therapy Control	34 32	n/a	67.5 ± 9.1	100.0	730.0 ± 628.0	severe	PANSS	positive, negative, general psychopathology	Prospective, non-RCT	Japan
Yang, W.Y. (1998)^7^	CCMD-2	Schizophrenia	Music therapy Control	40 30	2.4 3.2	38.7 ± 7.8	41.4	n/a	mild	BPRS SANS	negative symptoms	Prospective, RCT	China
Pavlicevic, M. (1994)^34^	SADS	Schizophrenia	Music therapy Control	21 20	9.5 0.0	38.2 ± 8.8	19.5	n/a	mild	BPRS SANS	negative symptoms	Prospective, non-RCT	UK
Tang, W. (1994)^6^	DSM-III-R	Schizophrenia, residual type	Music therapy Control	38 38	0.0 0.0	33.5 ± 8.0	19.7	531.0 ± 226.4	n/a	SANS	negative symptoms	Prospective, SB, RCT	China

Data presentation: mean ± SD

Abbreviation: *SD* standard deviation, *n*/*a* not available, *DSM*-*IV* diagnostic and statistical manual of mental disorders, 4th Edition, *DSM*-*IV*-*TR* diagnostic and statistical manual of mental disorders, 4th edition, text revision, *DSM*-*III*-*R* diagnostic and statistical manual of mental disorders, 3rd edition, revision, *ICD*-10 international classification of disease, 10th edition, *CCMD*-2 chinese classification of mental disorders, 2nd edition, *CCMD*-3, chinese classification of mental disorders, 3rd edition, *SADS* schedule for affective disorders and schizophrenia, *PANSS* positive and negative syndrome scales, *BPRS* brief psychiatric rating scales, *SANS* schedule for assessment of negative symptoms, *SDS* self-rating depression scales, *RCT* randomized controlled trials, *non*-*RCT* non randomized controlled trials, *DB* double-blinded, *SB* single-blinded, *UK* United Kingdom

### The main results of the current meta-analysis

**Fig. 2:**
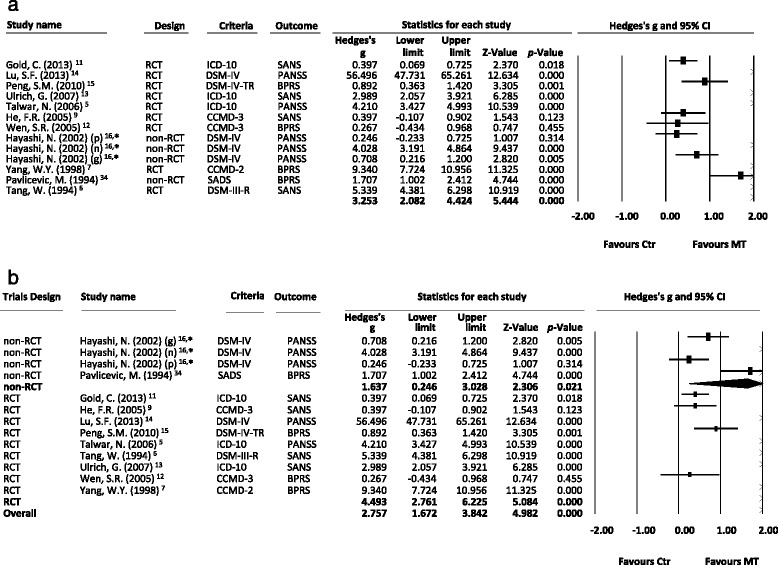
**a**. Forest plot showing effect sizes (Hedges’ *g*) and 95 % confidence intervals (CIs) from individual studies and pooled results of all included studies comparing total psychopathology between patients with schizophrenia receiving music therapy (MT) and those who did not receive music therapy (Ctr); **b**. Forest plot showing effect sizes (Hedges’ *g*) and 95 % CIs from individual studies and pooled results comparing total psychopathology between patients with schizophrenia receiving MT and the Ctr group by trial design, such as non-randomized control trials (non-RCT) and randomized control trials (RCT). *subscales in the report by Hayashi (2002): positive symptoms (p), negative symptoms (n), and general psychopathology (p). **a** The treatment effect was better in the MT group than in the Ctr group (*p* < .001). **b** The treatment effect was better in the MT group than in the Ctr group in both non-RCT and RCT subgroups (*p* = .021 and < .001, respectively). Abbreviation: MA: meta-analysis; CI: confidence interval; MT: music therapy; Ctr: control groups as schizophrenic patients without music therapy; DSM-IV: Diagnostic and Statistical Manual of Mental Disorders, 4th Edition; DSM-IV-TR: Diagnostic and Statistical Manual of Mental Disorders, 4th Edition, Text Revision; DSM-III-R: Diagnostic and Statistical Manual of Mental Disorders, 3rd Edition, Revision; ICD-10: international classification of disease, 10th edition; CCMD-2: Chinese Classification of Mental Disorders, 2nd edition; CCMD-3: Chinese Classification of Mental Disorders, 3rd edition; SADS: Schedule for Affective Disorders and Schizophrenia; PANSS: positive and negative syndrome scales; BPRS: brief psychiatric rating scales; SANS: Schedule for Assessment of Negative Symptoms; RCT: Randomized Controlled Trials; non-RCT: non Randomized Controlled Trials

After the screening procedure, no reports used a placebo in the control group, and none of the subjects were drug-free. Therefore, in the meta-analysis, we included studies that compared the treatment effect in schizophrenic patients receiving the standard treatment with adjunctive music therapy and those without adjunctive music therapy. A total of 402 schizophrenic patients who received adjunct music therapy and 402 who did not were extracted from the 12 studies. The treatment effect in the schizophrenic patients was significantly better in those treated with adjunct music therapy than in those treated without adjunct music therapy (ES = 3.25, 95 % confidence interval (CI): 2.08 to 4.42, *p* < 0.001) (Fig. 2a). Significant heterogeneity within these studies was found (Q = 498.7, df = 12, *I*^*2*^ = 97.6 %, *p* < 0.001). In addition, significant publication bias was detected using Egger’s test (t = 7.25, df = 11, 2-tailed *p* < 0.001) and in visual examination of the funnel plot. Furthermore, in subgroup meta-analysis of the different trial designs, we found a significantly better treatment effect in the schizophrenic patients who received adjunct music therapy than in those who did not in both non-RCT and RCT subgroups (ESs = 1.64, 95 % CI: 0.25 to 3.03, *p* = 0.021; ESs = 4.49, 95 % CI: 2.76 to 6.23, *p* < 0.001, respectively) (Fig. 2b).

We then used meta-regression analysis to investigate any possible confounding clinical variables within the studies. The results revealed that only the whole duration of illness and the number of previous hospitalizations were significantly associated with the treatment effect (slope = 3.30, *p* < 0.001; slope = −3.65, *p* = 0.04, respectively), but not mean age, gender (female proportion), frequency of music therapy, total duration of music therapy, duration of each session of music therapy, total number of sessions of music therapy, and chlorpromazine equivalence (*p* = 0.71, 0.40, 0.82, 0.36, 0.58, 0.79, and 0.46, respectively) (Table 2).

**Table 2:** Result of meta-regression of clinical variables and characters of MT with the treatment effect of adjunct MT in schizophrenic patients

Items	slope	*p* value
Mean age	0.11	0.712
female proportion	−0.10	0.404
Frequency of MT	−0.44	0.822
Total duration of MT	−0.73	0.362
Duration of each session of MT	6.61	0.576
Total number of sessions of MT	−0.06	0.789
Whole duration of illness***	3.30	<0.001
Chlorpromazine equivalence	−0.04	0.456
Number of previous hospitalization*	−3.65	0.040

Meta-regression with unrestricted maximum likelihood method

*: *p* < 0.05

***: *p* < 0.001

Abbreviation: *MT*: music therapy

**Fig. 3:**
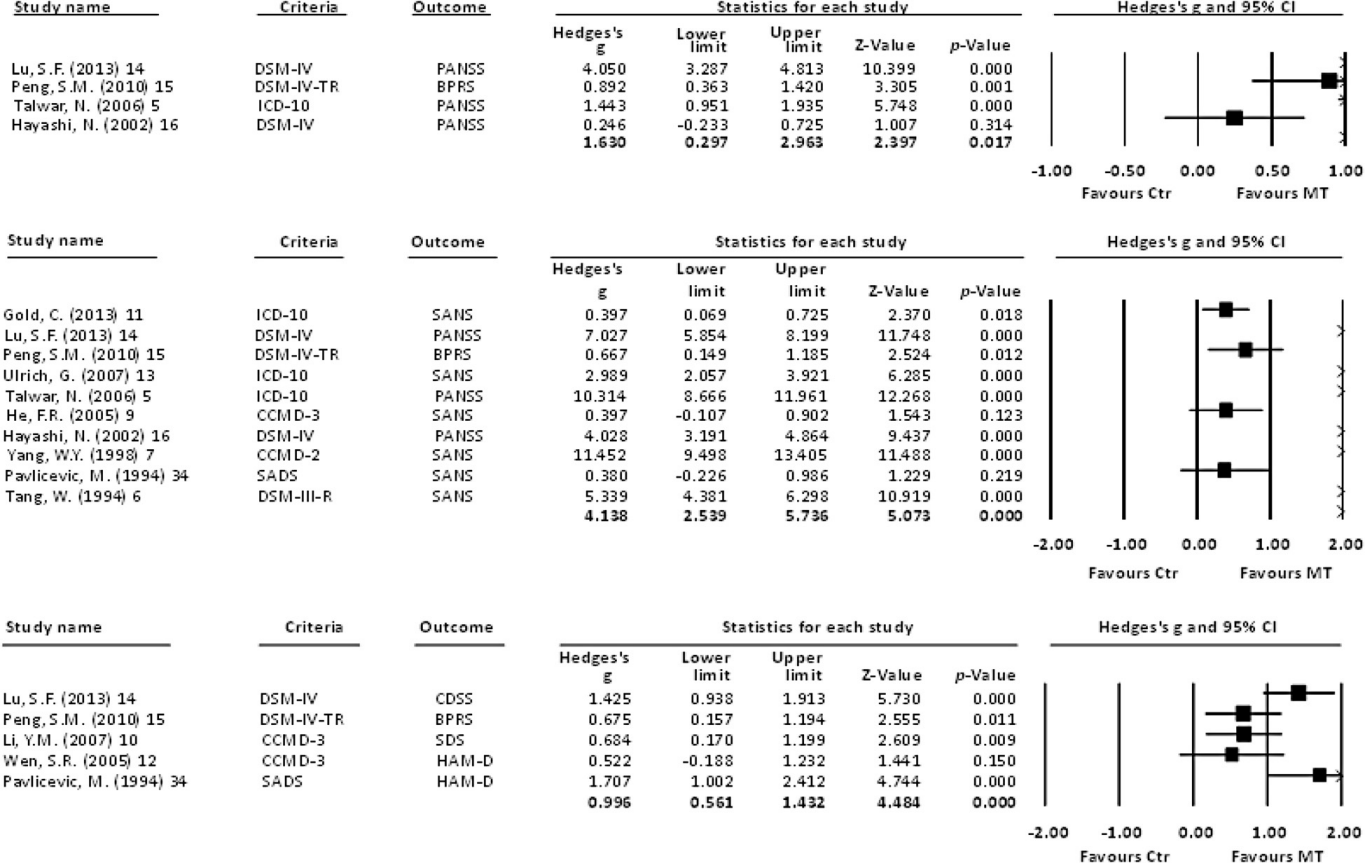
Forest plot showing effect sizes (Hedges’ *g*) and 95 % confident intervals (CIs) from individual studies and pooled results comparing **a**. positive symptoms, **b**. negative symptoms, and **c**. mood symptoms between schizophrenic patients who received music therapy (MT) and those who did not (Ctr). **a** The treatment effect was better in the MT group compared to the Ctr group in subscales of positive symptoms (*p* = .017). **b**. The treatment effect was better in the MT group compared to the Ctr group in subscales of negative symptoms (*p* < .001). **c**. The treatment effect was better in the MT group compared to the Ctr group in subscales of mood symptoms (*p* < .001). Abbreviation: MA: meta-analysis; CI: confidence interval; MT: music therapy; Ctr: control groups as schizophrenic patients without music therapy; DSM-IV: Diagnostic and Statistical Manual of Mental Disorders, 4th Edition; DSM-IV-TR: Diagnostic and Statistical Manual of Mental Disorders, 4th Edition, Text Revision; DSM-III-R: Diagnostic and Statistical Manual of Mental Disorders, 3rd Edition, Revision; ICD-10: international classification of disease, 10th edition; CCMD-2: Chinese Classification of Mental Disorders, 2nd edition; CCMD-3: Chinese Classification of Mental Disorders, 3rd edition; SADS: Schedule for Affective Disorders and Schizophrenia; PANSS: positive and negative syndrome scales; BPRS: brief psychiatric rating scales; SANS: Schedule for Assessment of Negative Symptoms; CDSS: depression scale for schizophrenia; SDS: Self-rating Depression Scales; HAM-D: Hamilton rating scale for depression

We then investigated whether or not music therapy was beneficial in any subset of psychiatric or mood symptoms. The results showed that music therapy improved positive symptoms (ES = 1.63, 95 % CI: 0.30 to 2.96, *p* = 0.017) (Fig. 3 (a)), negative symptoms (ES = 4.14, 95 % CI: 2.54 to 5.74, *p* < 0.001) (Fig. 3 (b)), and mood symptoms (ES = 1.00, 95 % CI: 0.56 to 1.43, *p* < 0.001) (Fig. 3 (c)), but not the subscales of general psychopathology (ES = 9.30, 95 % CI: −0.68 to 19.28, *p* = 0.068). We did not find any differences in dropout rate between the patients who did and did not receive adjunct music therapy (ES = −0.08, 95 % CI: −0.35 to 0.19, *p* = 0.574).

### The main results of subgroup meta-analysis in the duration of each session of music therapy

Although there was no significant association between the treatment effect of music therapy and the duration of each session of music therapy, we performed subgroup analysis by separating the included studies into those with a duration of less than 1 h [5, 11, 13, 24, 26] and those with a duration of 1 h or longer [6, 7, 23, 25]. The results revealed that both subgroups were associated with significantly better treatment effects in the patients who received adjunct music therapy than in those who did not (ES = 2.00, 95 % CI: 0.70 to 3.31, *p* = 0.003; ES = 7.62, 95 % CI: 4.66 to 10.58, *p* < 0.001, respectively) (Table 3).

**Table 3:** Result of subgroup meta-analysis of the treatment effect of adjunct MT in schizophrenic patients

Subgroup MA		Effect sizes	95 % confidence interval		*p* value
			Lower limit	Upper limit	
Duration of each session of MT	<1 h	2.00	0.70	3.31	0.003
	> or = 1 h	7.62	4.66	10.58	<0.001
Total duration of MT	<3 months	3.97	2.03	5.91	<0.001
	> or = 3 months	3.00	1.36	4.64	<0.001
Frequency of MT	<4 times/week	3.40	1.86	4.93	<0.001
	> or = 4 times/week	3.13	0.95	5.31	0.005
Total number of sessions of MT	<20 sessions	3.92	2.32	5.52	<0.001
	> or = 20 session	2.33	0.50	4.17	0.013
Different disease severity	Mild	7.34	4.50	10.18	<0.001
	Moderate	n/a	n/a	n/a	n/a
	Severe	n/a	n/a	n/a	n/a

Abbreviation: *MT* music therapy, *MA* meta-analysis, *n*/*a* no applicable

### The main results of subgroup meta-analysis in the total duration of music therapy

Similarly, we performed subgroup analysis by separating the included studies into those with a total duration of music therapy shorter than 3 months [6, 9, 12, 13, 23, 24, 26] and those with a total duration of 3 months or longer [5, 7, 11, 25] according to guidelines used in a previous report [15]. The results revealed that both subgroups were associated with a significantly better treatment effect in the patients who received adjunct music therapy than in those who did not (ES = 3.97, 95 % CI: 2.03 to 5.91, *p* < 0.001; ES = 3.00, 95 % CI: 1.36 to 4.64, *p* < 0.001, respectively) (Table 3).

### The main results of subgroup meta-analysis in the frequency of music therapy

We then performed subgroup analysis by separating the included studies into those with a frequency of music therapy less than 4 times per week [5, 11, 13, 23, 25, 26] and those with a frequency of 4 times or more per week [6, 7, 9, 12, 24], because of the significant positive association between the treatment effect of music therapy and the frequency of music therapy. Both frequencies provided a significantly better treatment effect in the schizophrenic patients who received adjunct music therapy than in those who did not (ES = 3.40, 95 % CI: 1.86 to 4.93, *p* < 0.001; ES = 3.13, 95 % CI: 0.95 to 5.31, *p* = 0.005, respectively) (Table 3).

### The main results of subgroup meta-analysis in the total number of sessions of music therapy

We then performed subgroup analysis by separating the included studies into those with less than 20 sessions in total [5, 6, 13, 23–26] and with 20 sessions or more [7, 9, 11, 12] according to a previous report [15]. The results revealed that both subgroups were associated with a significantly better treatment effect in the patients who received adjunct music therapy than in those who did not (ES = 3.92, 95 % CI: 2.32 to 5.52, *p* < 0.001; ES = 2.33, 95 % CI: 0.50 to 4.17, *p* = 0.013, respectively) (Table 3).

### The main results of the subgroup meta-analysis of those with different subgroups of disease severity

We then subdivided the studies according to their mean scores of disease severity, either in the PANSS or BPRS. The criteria of disease severity were based on the report by Leucht et al. [27, 28], which defined “mildly ill” as a PANSS score of 58 or BPRS 31, “moderately ill” as PANSS 75 or BPRS 41, and “severely ill” as PANSS 95 or BPRS 53. In the current meta-analysis, only one study recruited schizophrenic patients with severe disease severity [25], one with moderate disease severity [12], and another two with unknown disease severity which did not provide definite scores of the rating scales [6, 11]. All of the other reports included subjects with mild disease severity. Therefore, we could only perform subgroup meta-analysis in those with mild disease severity [5, 7, 13, 23, 24, 26]. However, the results still revealed a significantly better treatment effect (ES = 7.34, 95 % CI: 4.50 to 10.18, *p* < 0.001) (Table 3).

### The main results of the subgroup meta-analysis of different strategy of music therapy

We then subdivided the studies according to the strategy of music therapy applied in their studies. At first, we subdivided them according to forms of music therapy, including forms of group therapy [6, 9, 10, 12, 13, 23–26], individual therapy [5, 11], or both [7]. Therefore, we could perform subgroup meta-analysis only in subgroup of forms of group therapy, which revealed similar results (ES = 3.62, 95 % CI: 2.21 to 5.02, *p* < 0.001). In the other hand, we subdivided them according to the activities in the sessions of music therapy, including those with active participation only, passive listening only [6, 7], or both [5, 9–13, 23–26]. Therefore, we could perform subgroup meta-analysis only in subgroup of studies using sessions of music therapy including both active participation and passively listening, which revealed similar results again (ES = 2.26, 95 % CI: 1.20 to 3.32, *p* < 0.001).

## Discussion

The results of this meta-analysis indicated that adjunct music therapy to standard treatment was associated with a significantly better treatment effect in schizophrenic patients than control patients, not only in negative or mood symptoms but also in positive symptoms. In addition, this treatment effect was significantly positively associated with the whole duration of illness, and significantly inversely associated with the number of previous hospitalizations.

In general, the results of this study are consistent with the previous meta-analysis conducted by Mössler et al. (2011) [15], which report moderate to large effects in general mental state, negative symptoms, depression, and anxiety. In addition, the current meta-analysis provides additional information to the current knowledge about the benefits of adjunct music therapy for patients with schizophrenia, which provided the evidences of the significant treatment effect of adjunctive music therapy on the positive symptoms, negative symptoms, and mood symptoms. In the meta-analysis conducted by Mössler et al. (2011), the treatment effect of music therapy on positive symptoms was inconclusive because of limited evidence. In recent years, several new trials and studies have been published [11, 23, 24]. Among them, most studies discussed the significantly better treatment effect of adjunct music therapy on the positive symptoms in schizophrenic patients [23, 24]. The significantly better treatment effect in newer published studies rather than previous reports might be derived from the different intervention design (group therapy versus individual therapy) [5, 23], different frequency of music therapy [24, 25], or different total duration of music therapy [5, 24]. Thus, it is necessary to conduct a further meta-analysis based on these newer evidences. The clinical implication that adjunct music therapy can alleviate positive symptoms in schizophrenic patients more effectively than in those who do not receive adjunct music therapy is important, in particular because positive symptoms are thought to be highly associated with the risk of violence [2]. In addition, Courtright P and the colleague (1990) reported that the use of music therapy could help in reducing aggressive behavior in schizophrenic patients [29]. Among the positive symptoms, the hallucination and delusion had been thought to be more prominent in clinical practice. Furthermore, the relationship between the auditory hallucination and music therapy was also one of the interesting topics about the treatment effect of adjunct music therapy in schizophrenic patients. However, we could not perform such meta-analysis of these topics because of the limited reports providing these data [7, 24]. Among these articles, there was only one discussing the differences of severity of hallucination or delusion after treatment of adjunct music therapy [7] and only one comparing the changes of severity of hallucination or delusion between experiment group (patients with adjunct music therapy) and control group (patients with standard treatment only) [24]. When we investigated the results of these two studies, we found significantly lower severity of hallucination and delusion after treatment of adjunct music therapy and significantly better improvement in hallucination in patients with adjunct music therapy than those with standard treatment only but not in delusion.

We also performed meta-regression analysis to evaluate the possible confounding effect of clinical variables. The results showed that the treatment effect of music therapy was positively associated with the “whole duration of illness”. This means that the application of music therapy for schizophrenic patients could alleviate disease severity, especially in those with chronic illness. Several authors have investigated the effect of music therapy on cognitive function in patients with chronic schizophrenia, and reported significant benefits [30]. In addition, the usage of specific music therapy has been reported to improve attention and motor performance in chronic schizophrenic patients [8, 31, 32]. Taken together, supplemental music therapy can provide significant benefits for schizophrenic patients with a chronic course with regards to disease severity and also cognitive function.

In this study, we found a significantly inverse association between the treatment effect of music therapy and the number of previous hospitalizations. This seems to be in contrast to our other finding of a positive association between the treatment effect of music therapy and the whole duration of illness. A possible explanation may be the specific characteristics of the patients with more previous hospitalizations. In previous reports, schizophrenic patients with more previous hospitalizations have been reported to have worse treatment adherence, poorer treatment attitude, easier relapse, and poor support systems [33–35]. Therefore, the adherence or attendance rate to music therapy in schizophrenic patients with more previous hospitalizations may be poorer than those with fewer previous hospitalizations. However, we could not investigate the association between adherence/attendance rate and the treatment effect of music therapy because of a lack of data.

With regards to safety profile, the dropout rate was not significantly different between the schizophrenic patients who did and not receive adjunct music therapy. In addition, when we investigated the included studies individually, no severe adverse effect profile was reported with the use of music therapy.

With regards to the cost-effectiveness of music therapy in the treatment of schizophrenic patients, we found no significant associations between the treatment effect of music therapy and the frequency of music therapy, the total duration of music therapy, and the duration of each session of music therapy in meta-regression analysis. Furthermore, we investigated whether there was any difference in the treatment effect of adjunct music therapy in subgroup analysis by different frequency of music therapy or different total duration of music therapy. The results showed that the treatment effect was significantly better compared to those who did not receive adjunct music therapy, both in a total duration of music therapy shorter than 3 months and 3 months or longer, and a frequency of less than 4 times per week and 4 times a week or more. This indicates that adjunct music therapy in schizophrenic patients can result in a better treatment effect compared to those who do not receive adjunct music therapy when introduced into a treatment program regardless of the total duration, frequency, or duration of each session of music therapy. Because music therapy is costly and complex, clinicians may be reluctant to include it in clinical practice. However, our findings show that the effect of music therapy is beneficial regardless of the frequency, length, and duration. Therefore, we suggest that clinicians should consider including adjunct music therapy into their clinical practice to provide their patients with the best outcomes possible.

### Limitation

There are some limitations to the current meta-analysis. First, most of the included studies conducted adjunct music therapy as a combination of passive listening and active participation and most of them were performed in forms of group therapy. This may lead to the problem that, for most of the studies that involved playing music, the therapy group would likely consist of patients who knew how to play an instrument. However, in the real world, this situation is unlikely to occur. Further studies are needed to investigate whether “passive listening” or “individual therapy” can provide benefits for schizophrenic patients, either in disease severity or with other symptoms. Second, as mentioned in the Discussion, we could not further analyze the association between the treatment effect of music therapy and the attendance rate of music therapy because of a lack of data. However, the attendance rate may be a confounding effect on the treatment effect of music therapy. Third, in order to perform the meta-analysis for the treatment effect of music therapy on the general aspect of disease severity, positive symptoms, negative symptoms, general psychopathology, and mood symptoms, we pooled different studies with different rating scales including the PANSS, BPRS, SANS, and SDS. This may have increased the heterogeneity in the meta-analysis, which achieved significance. However, in previous reports, disease severity was shown to be associated between different rating scales to some degree [27, 28]. In addition, there were no absolute contraindications to pool these different rating scales together. Therefore, in order thoroughly meta-analyze the subscales of specific symptoms, we chose to pool those rating scales together. Besides, in current study, we could not perform detailed meta-analysis of treatment effect of adjunct music therapy for the specific symptoms, such as hallucination or delusion, because of the limited reports providing such information. Finally, we tried to investigate the treatment effect of music therapy on schizophrenic patients with different disease severity. However, we could only perform the meta-analysis on those with mild disease severity but not those with moderate or severe disease severity because of a lack of sufficient trials.

## Conclusion

Our meta-analysis provides strong evidence to prove the role of adjunct music therapy in the treatment of schizophrenic patients. In these patients, adjunct music therapy improved the effect of treatment in negative and mood symptoms, and also positive symptoms compared to those who did not receive music therapy regardless of the total duration, frequency, total number of sessions, or the duration of each session of music therapy. The effect of treatment was especially pronounced for those with a chronic disease course. However, in order to extensively apply adjunct music therapy in a clinical setting, further trials are needed to evaluate the treatment effect of music therapy with “passive listening,” the possible confounding effect of attendance rate, and the treatment effect of music therapy on those with moderate or severe disease severity.

### Availability and requirements

The database used in current meta-analysis is PubMed, which website is http://www.ncbi.nlm.nih.gov/pubmed. In addition, there are not any restrictions to its use by non-academics.

## Additional files

Additional file 1: The PRISMA checklist of current meta-analysis. (DOCX 31 kb) Additional file 2: Table S1. Summary of Jadad scores of studies in current meta-analysis. (DOCX 15 kb)

## Abbreviations

BPRS: brief psychiatric rating scales
CCMD-2: chinese classification of mental disorders, 2nd edition
ccmd-3: chinese classification of mental disorders: 3rd edition
CDSS: depression scale for schizophrenia
CI: confidence interval
Ctr: control groups
DSM-III-R: diagnostic and statistical manual of mental disorders, 3rd edition, revision
DSM-IV: diagnostic and statistical manual of mental disorders, 4th edition
DSM-IV-TR: diagnostic and statistical manual of mental disorders, 4th edition, text revision
ESs: effect sizes
HAM-D: hamilton rating scale for depression
ICD-10: international classification of disease, 10th edition
MA: meta-analysis
MT: music therapy
non-RCT: non-randomized control trials
PANSS: positive and negative symptoms scales
PRISMA: preferred reporting items for systematic reviews and meta-analyses
RCT: randomized control trials
SADS: schedule for affective disorders and schizophrenia
SANS: scale for the assessment of negative symptoms
SDS: self-rating depression scales

## References

[1] Kay SR, Fiszbein A, Opler LA (1987). The positive and negative syndrome scale (PANSS) for schizophrenia. Schizophr Bull.

[2] Sands N, Elsom S, Gerdtz M, Khaw D (2012). Mental health-related risk factors for violence: using the evidence to guide mental health triage decision making. J Psychiatr Ment Health Nurs.

[3] Bagaric D, Brecic P, Ostojic D, Jukic V, Goles A (2013). The relationship between depressive syndrome and suicidal risk in patients with acute schizophrenia. Croat Med J.

[4] Challis S, Nielssen O, Harris A, Large M (2013). Systematic meta-analysis of the risk factors for deliberate self-harm before and after treatment for first-episode psychosis. Acta Psychiatr Scand.

[5] Talwar N, Crawford MJ, Maratos A, Nur U, McDermott O, Procter S (2006). Music therapy for in-patients with schizophrenia: exploratory randomised controlled trial. Br J Psychiatry.

[6] Tang W, Yao X, Zheng Z (1994). Rehabilitative effect of music therapy for residual schizophrenia. A one-month randomised controlled trial in Shanghai. Br J Psychiatry Suppl.

[7] Yang WY, Li Z, Weng YZ, Zhang HY, Ma B (1998). Psychosocial rehabilitation effects of music therapy in chronic schizophrenia. Hong Kong Journal of Psychiatry.

[8] Shih YN, Chen CS, Chiang HY, Liu CH (2014). Influence of background music on work attention in clients with chronic schizophrenia. Work.

[9] He FR, Liu RK, Ma L (2005). Influence of musical therapy on serum PRL of patients with schizophrenia, type II [yin yue xin li zhi liao dui xing jing shen fen lie huan zhe xue qing cui ru su shui ping de ying xiang]. Shandong Archives of Psychiatry.

[10] Li YM, Ren X, Li CP, Li ZQ (2007). The correct effect of language guided music therapy on patients with schizophrenia [yu yan you dao shi yin yue liao fa dui jing shen fen lie huan zhe de xin li jiao zhi xiao guo]. International Nurses Journal.

[11] Gold C, Mossler K, Grocke D, Heldal TO, Tjemsland L, Aarre T (2013). Individual music therapy for mental health care clients with low therapy motivation: multicentre randomised controlled trial. Psychother Psychosom.

[12] Wen SR, Cao GY, Zhou HS (2005). The effect of music therapy on the depressive position of patients with schizophrenia [yin yue zhi liao dui jing shen fen lie huan zhe de yi yu zhuang tai de ying xiang]. Chinese Journal of Clinical Rehabilitation.

[13] Ulrich G, Houtmans T, Gold C (2007). The additional therapeutic effect of group music therapy for schizophrenic patients: a randomized study. Acta Psychiatr Scand.

[14] Kamioka H, Tsutani K, Yamada M, Park H, Okuizumi H, Tsuruoka K (2014). Effectiveness of music therapy: a summary of systematic reviews based on randomized controlled trials of music interventions. Patient Prefer Adherence.

[15] Mossler K, Chen X, Heldal TO, Gold C (2011). Music therapy for people with schizophrenia and schizophrenia-like disorders. Cochrane Database Syst Rev.

[16] Dubugras MT, Evans-Lacko S, Mari JJ (2011). A two-year cross-sectional study on the information about schizophrenia divulged by a prestigious daily newspaper. J Nerv Ment Dis.

[17] Jadad AR, Moore RA, Carroll D, Jenkinson C, Reynolds DJ, Gavaghan DJ (1996). Assessing the quality of reports of randomized clinical trials: is blinding necessary?. Control Clin Trials.

[18] Overall JE (1988). The Brief Psychiatric Rating-Scale (Bprs) - Recent Developments in Ascertainment and Scaling - Introduction. Psychopharmacol Bull.

[19] Andreasen NC (1989). The Scale for the Assessment of Negative Symptoms (SANS): conceptual and theoretical foundations. Br J Psychiatry Suppl.

[20] Zung WW (1965). A Self-Rating Depression Scale. Arch Gen Psychiatry.

[21] Egger M, Davey Smith G, Schneider M, Minder C (1997). Bias in meta-analysis detected by a simple, graphical test. BMJ.

[22] Liberati A, Altman DG, Tetzlaff J, Mulrow C, Gotzsche PC, Ioannidis JP (2009). The PRISMA statement for reporting systematic reviews and meta-analyses of studies that evaluate health care interventions: explanation and elaboration. PLoS Med.

[23] Lu SF, Lo CH, Sung HC, Hsieh TC, Yu SC, Chang SC (2013). Effects of group music intervention on psychiatric symptoms and depression in patient with schizophrenia. Complement Ther Med.

[24] Peng SM, Koo M, Kuo JC (2010). Effect of group music activity as an adjunctive therapy on psychotic symptoms in patients with acute schizophrenia. Arch Psychiatr Nurs.

[25] Hayashi N, Tanabe Y, Nakagawa S, Noguchi M, Iwata C, Koubuchi Y (2002). Effects of group musical therapy on inpatients with chronic psychoses: a controlled study. Psychiatry Clin Neurosci.

[26] Pavlicevic M, Trevarthen C, Duncan J (1994). Improvisational Music-Therapy and the Rehabilitation of Persons Suffering from Chronic-Schizophrenia. J Music Ther.

[27] Leucht S, Kane JM, Kissling W, Hamann J, Etschel E, Engel R (2005). Clinical implications of Brief Psychiatric Rating Scale scores. Br J Psychiatry.

[28] Leucht S, Kane JM, Kissling W, Hamann J, Etschel E, Engel RR (2005). What does the PANSS mean?. Schizophr Res.

[29] Courtright P, Johnson S, Baumgartner MA, Jordan M, Webster JC (1990). Dinner music: does it affect the behavior of psychiatric inpatients?. J Psychosoc Nurs Ment Health Serv.

[30] Kwon M, Gang M, Oh K (2013). Effect of the Group Music Therapy on Brain Wave, Behavior, and Cognitive Function among Patients with Chronic Schizophrenia. Asian Nurs Res (Korean Soc Nurs Sci).

[31] Glicksohn J, Cohen Y (2000). Can music alleviate cognitive dysfunction in schizophrenia?. Psychopathology.

[32] Chambliss C, McMichael H, Tyson K, Monaco C, Tracy J (1996). Motor performance of schizophrenics after mellow and frenetic antecedent music. Percept Mot Skills.

[33] Eticha T, Teklu A, Ali D, Solomon G, Alemayehu A (2015). Factors associated with medication adherence among patients with schizophrenia in Mekelle, Northern Ethiopia. PLoS One.

[34] Shao WC, Chen H, Chang YF, Lin WC, Lin EC (2013). The relationship between medication adherence and rehospitalization: a prospective study of schizophrenia patients discharged from psychiatric acute wards. Hu Li Za Zhi.

[35] San L, Bernardo M, Gomez A, Martinez P, Gonzalez B, Pena M (2013). Socio-demographic, clinical and treatment characteristics of relapsing schizophrenic patients. Nord J Psychiatry.

