# Erratum to: Significant treatment effect of adjunct music therapy to standard treatment on the positive, negative, and mood symptoms of schizophrenic patients: a meta-analysis

**DOI:** 10.1186/s12888-016-0846-1

**Published:** 2016-05-17

**Authors:** Ping-Tao Tseng, Yen-Wen Chen, Pao-Yen Lin, Kun-Yu Tu, Hung-Yu Wang, Yu-Shian Cheng, Yi-Chung Chang, Chih-Hua Chang, Weilun Chung, Ching-Kuan Wu

**Affiliations:** Department of Psychiatry, Tsyr-Huey Mental Hospital, Kaohsiung Jen-Ai’s Home, Taiwan, No.509, Fengping 1st Rd., Daliao Dist, Kaohsiung, 831 Taiwan; Department of Neurology, E-Da Hospital, Kaohsiung, Taiwan; Department of Psychiatry, Kaohsiung Chang Gung Memorial Hospital and Chang Gung University College of Medicine, Kaohsiung, Taiwan; Center for Translational Research in Biomedical Sciences, Kaohsiung Chang Gung Memorial Hospital, Kaohsiung, Taiwan

In article “Significant treatment effect of adjunct music therapy to standard treatment on the positive, negative, and mood symptoms of schizophrenic patients: a meta-analysis [1]”, some values of Hedges’ g in main results of the meta-analysis might mislead the readers’ interpretation of our results. Different values of Hedges’ g may derive from different methods of standardization when using Comprehensive Meta-analysis software. The main results of significance in current study remained significant. By using different method of standardization, we found that the main treatment effect of adjunct music therapy in schizophrenia was significantly larger than those without adjunct music therapy (Hedges’ g = 0.596, 95 % CI = 0.350-0.842, *p* < 0.001). At the same time, the treatment effect of adjunct music therapy in schizophrenia remained significantly larger than those without adjunct music therapy in scores of positive symptoms, negative symptoms, and mood symptoms (Hedges’ g = 0.483, 95 % CI = 0.053-0.913, *p* = 0.028; Hedges’ g = 0.673, 95 % CI = 0.385-0.961, *p* < 0.001; Hedges’ g = 0.677, 95 % CI = 0.434-0.919, *p* < 0.001, separately).

**Fig. 2:**
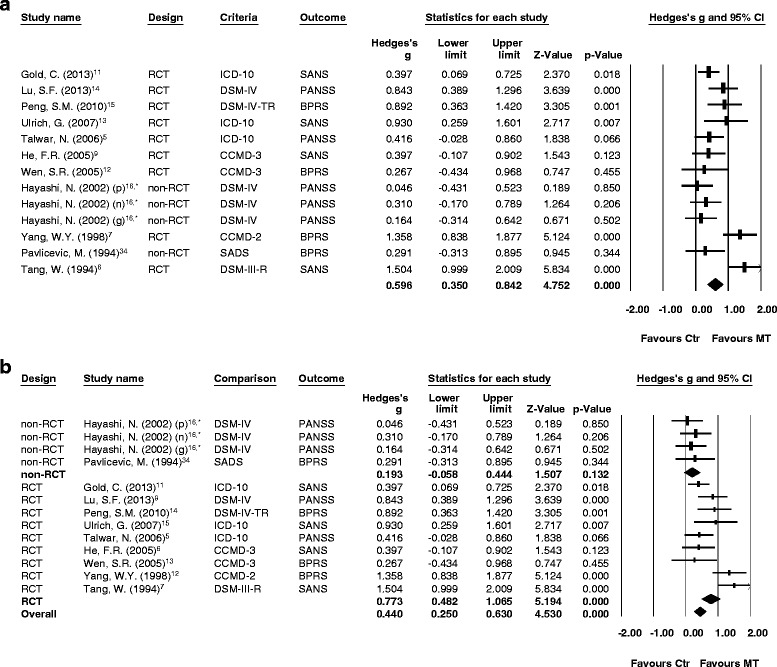
**a** Forest plot showing effect sizes (Hedges’ *g*) and 95 % confidence intervals (CIs) from individual studies and pooled results of all included studies comparing total psychopathology between patients with schizophrenia receiving music therapy (MT) and those who did not receive music therapy (Ctr); (**b**) Forest plot showing effect sizes (Hedges’ *g*) and 95 % CIs from individual studies and pooled results comparing total psychopathology between patients with schizophrenia receiving MT and the Ctr group by trial design, such as non-randomized control trials (non-RCT) and randomized control trials (RCT). *subscales in the report by Hayashi (2002): positive symptoms (*p*), negative symptoms (*n*), and general psychopathology (g). (A) The treatment effect was better in the MT group than in the Ctr group (*p* < 0.001). (B) The treatment effect was better in the MT group than in the Ctr group in both non-RCT and RCT subgroups (*p* = 0.132 and <0.001, respectively)

**Fig. 3:**
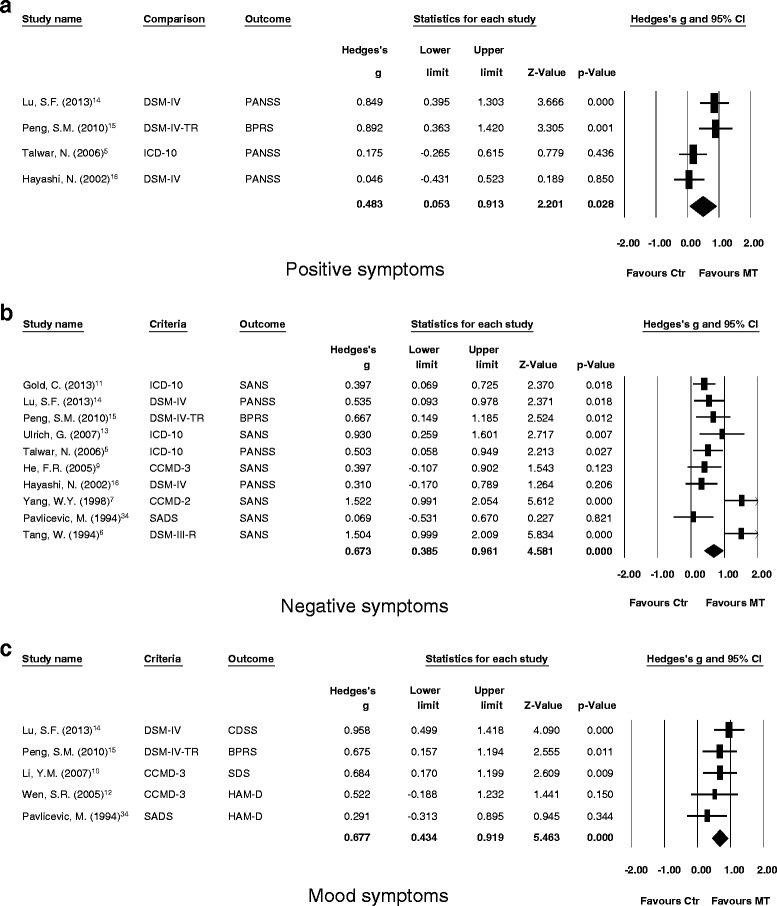
Forest plot showing effect sizes (Hedges’ *g*) and 95 % confident intervals (CIs) from individual studies and pooled results comparing (**a**) positive symptoms, (**b**) negative symptoms, and (**c**) mood symptoms between schizophrenic patients who received music therapy (MT) and those who did not (Ctr). (A) The treatment effect was better in the MT group compared to the Ctr group in subscales of positive symptoms (*p* = 0.028). (B) The treatment effect was better in the MT group compared to the Ctr group in subscales of negative symptoms (*p* < 0.001). (C) The treatment effect was better in the MT group compared to the Ctr group in subscales of mood symptoms (*p* < 0.001)
