# Supplementary material for: Significant treatment effect of adjunct music therapy to standard treatment on the positive, negative, and mood symptoms of schizophrenic patients: a meta-analysis
Source: BMC Psychiatry. 2016 Jan 26;16:16. doi: 10.1186/s12888-016-0718-8 (PMC4728768; doi:10.1186/s12888-016-0718-8)
Supplement: Additional file 2: Table S1. — Summary of Jadad scores of studies in current meta-analysis. (DOCX 15 kb) [file 12888_2016_718_MOESM2_ESM.docx]

| Study | Randomization | Blinding | Account of all patients | Total Jadad scores |
| --- | --- | --- | --- | --- |
| Gold, C. (2013)^11^ | 2 | 0 | 1 | 3 |
| Lu, S.F. (2013)^14^ | 2 | 0 | 1 | 3 |
| Peng, S.M. (2010)^15^ | 1 | 0 | 0 | 1 |
| Li, Y.M. (2007)^10^ | 1 | 0 | 0 | 1 |
| Ulrich, G. (2007)^13^ | 1 | 2 | 1 | 4 |
| Talwar, N. (2006)^5^ | 2 | 0 | 0 | 2 |
| He, F.R. (2005)^9^ | 1 | 0 | 0 | 1 |
| Wen, S.R. (2005)^12^ | 1 | 0 | 0 | 1 |
| Hayashi, N. (2002)^16^ | 0 | 0 | 0 | 0 |
| Yang, W.Y. (1998)^7^ | 1 | 0 | 0 | 1 |
| Pavlicevic, M. (1994)^34^ | 0 | 0 | 0 | 0 |
| Tang, W. (1994)^6^ | 1 | 0 | 1 | 2 |

**Additional file 2: Table S1. Summary of Jadad scores of studies in current meta-analysis**
